# Trends in hypofractionated radiotherapy use among patients with breast or prostate cancer: a multicenter study in Osaka, Japan

**DOI:** 10.1093/jrr/rraf082

**Published:** 2026-01-13

**Authors:** Toshiki Ikawa, Toshitaka Morishima, Kayo Nakata, Yoshihiro Kuwabara, Kenji Kishimoto, Naoyuki Kanayama, Masahiro Morimoto, Koji Konishi, Isao Miyashiro

**Affiliations:** Cancer Control Center, Osaka International Cancer Institute, 3-1-69 Otemae, Chuo-ku, Osaka 541-8567, Japan; Department of Radiation Oncology, Osaka International Cancer Institute, 3-1-69 Otemae, Chuo-ku, Osaka 541-8567, Japan; Cancer Control Center, Osaka International Cancer Institute, 3-1-69 Otemae, Chuo-ku, Osaka 541-8567, Japan; Cancer Control Center, Osaka International Cancer Institute, 3-1-69 Otemae, Chuo-ku, Osaka 541-8567, Japan; Cancer Control Center, Osaka International Cancer Institute, 3-1-69 Otemae, Chuo-ku, Osaka 541-8567, Japan; Cancer Control Center, Osaka International Cancer Institute, 3-1-69 Otemae, Chuo-ku, Osaka 541-8567, Japan; Department of Radiation Oncology, Osaka International Cancer Institute, 3-1-69 Otemae, Chuo-ku, Osaka 541-8567, Japan; Department of Radiation Oncology, Osaka International Cancer Institute, 3-1-69 Otemae, Chuo-ku, Osaka 541-8567, Japan; Department of Radiation Oncology, Osaka International Cancer Institute, 3-1-69 Otemae, Chuo-ku, Osaka 541-8567, Japan; Cancer Control Center, Osaka International Cancer Institute, 3-1-69 Otemae, Chuo-ku, Osaka 541-8567, Japan

**Keywords:** breast neoplasms, hypofractionation, patterns, prostate neoplasms, trends

## Abstract

The uptake of hypofractionated radiotherapy in Japan is not well documented. This study examined trends in the proportion of patients with breast or prostate cancer who received hypofractionated radiotherapy, using hospital-based cancer registry data linked to Diagnostic Procedure Combination records from 69 institutions in Osaka Prefecture (diagnosis years: 2019–23). Eligible patients included 6475 women with unilateral breast cancer (pTisN0M0, pT1–2N0M0 or cT1–2N0M0 before neoadjuvant therapy) who underwent partial mastectomy and radiotherapy at the same hospital and 3274 men with cT1–3N0M0 prostate cancer treated with external-beam radiotherapy without surgery or brachytherapy. The use of hypofractionated radiotherapy was determined through insurance claims. Among the patients with breast cancer, the proportion of those who received hypofractionated radiotherapy increased from 41% in 2019 to 81% in 2023. In 2023, this proportion was highest at facilities with high radiotherapy volume (95%), followed by those with medium (84%) and low (67%) volumes. Among the patients with prostate cancer, the proportion of those who received hypofractionated radiotherapy (hypofractionated intensity-modulated radiotherapy or stereotactic body radiotherapy) increased from 25% in 2019 to 48% in 2023, although the increase slowed after 2021. In 2023, hypofractionated radiotherapy use reached 79% at high-volume facilities, while proportions were lower at medium- (30%) and low-volume (17%) facilities. Hypofractionated radiotherapy use has increased in patients with breast and prostate cancers. However, its adoption in prostate cancer treatment remains limited, particularly in medium- and low-volume facilities. These findings suggest that certain barriers limit its implementation and highlight the need to address them.

## INTRODUCTION

Radiotherapy is one of the principal modalities used in cancer treatment and is typically delivered in fractionated doses on consecutive weekdays. For example, postoperative radiotherapy after breast-conserving surgery is conventionally delivered at a total dose of 45–50.4 Gy in 25–28 fractions over ~5 weeks [1]. These prolonged treatment courses place a substantial burden on patients and treatment facilities. In the treatment of breast cancer following breast-conserving surgery and of localized prostate cancer, clinical trials have shown that hypofractionated regimens, which involve fewer fractions and higher doses per fraction, achieve outcomes comparable to conventionally fractionated radiotherapy [2–4]. Evidence-based guidelines support the use of hypofractionation [1, 5, 6], and its adoption is increasing [7–9]. Furthermore, the coronavirus disease 2019 (COVID-19) pandemic, which strained healthcare systems worldwide, may have further promoted its adoption [10, 11].

However, the adoption of hypofractionated radiotherapy (HF-RT) is influenced by local reimbursement systems, and utilization patterns have been reported to differ across countries [12, 13]. In countries that use a fee-for-service reimbursement model—in which payments are made per fraction delivered—the reimbursement structure may act as a major barrier to the adoption of HF-RT owing to reduced hospital revenue [12–14]. Therefore, assessing the current uptake of HF-RT in each region is essential for informing local policy decisions.

Japan has universal health coverage, and reimbursements to healthcare providers are regulated by a nationally uniform fee schedule [15]. External beam radiotherapy, excluding stereotactic techniques, is paid per fraction. For breast and prostate cancers, an additional fee is provided for hypofractionated regimens that use a defined higher dose per fraction. Previous studies using the Japanese National Database have reported an increase in the number of courses and fractions delivered as HF-RT for breast and prostate cancers following the COVID-19 pandemic [11, 16]. However, these claims-based analyses lack tumor-node-metastasis (TNM) classification and stage information, limiting the ability to estimate the proportion of patients receiving HF-RT among eligible patients.

To address this limitation, this study analyzed trends in the use of HF-RT among patients with breast or prostate cancer, using hospital-based cancer registry data linked to Diagnosis Procedure Combination (DPC) records. Furthermore, factors associated with the use of HF-RT were examined.

## MATERIALS AND METHODS

### Study design and population

This observational study was conducted as part of the cancer registry-based study on cancer care in Osaka (CanReCO), a project led by the Council for the Coordination of Designated Cancer Care Hospitals in Osaka. The CanReCO includes hospital-based cancer registry data linked to DPC records from all nationally designated (*n* = 18) and prefecture-designated (*n* = 51) cancer care hospitals—whether currently or previously designated—in Osaka Prefecture, the third-most populous prefecture in Japan (population, 8.8 million in 2023). These data cover ~80–90% of surgical or endoscopic resection cases for cancer in the region [17].

The hospital-based cancer registries provided information on patients’ sex; age at diagnosis; date of diagnosis; anonymized hospital code; tumor site, histology and behavior code based on the International Classification of Diseases for Oncology, 3rd edition (ICD-O-3); clinical and pathological TNM classification (Union for International Cancer Control, 8th edition); laterality; prostate cancer grade group; and receipt of surgery, radiotherapy, hormone therapy or chemotherapy (including targeted agents and immunotherapy) as initial treatment. DPC records contain claims data on inpatient and outpatient services, including surgical procedures such as partial or total mastectomy, radiotherapy techniques such as intensity-modulated radiotherapy (IMRT) and stereotactic body radiotherapy (SBRT) and add-on fees for hypofractionation.

Patients diagnosed between 2019 and 2023 were eligible for inclusion if they met all of the following criteria: a site code of C50 (breast) or C61 (prostate); a histology code classified as an epithelial tumor according to the Rare Cancer Classification table from the SEER Program [18]; a behavior code of 2 (*in situ*) or 3 (malignant) for breast cancer and 3 for prostate cancer; and underwent initial treatment at the registered institution. Patients with breast cancer were limited to women with unilateral pTisN0M0 or pT1–2N0M0 tumors, or, when neoadjuvant therapy precluded pathologic staging, cT1–2N0M0 tumors, all of whom underwent partial mastectomy and external beam radiotherapy at the same hospital. Patients with prostate cancer were limited to those with cT1–3N0M0 tumors who underwent external beam radiotherapy without surgery or brachytherapy.

For breast cancer, radiotherapy was classified as conventionally fractionated or hypofractionated based on the presence of an add-on fee for hypofractionation (doses ≥2.5 Gy per fraction for whole-breast irradiation). Because the add-on fee cannot be claimed when radiotherapy is delivered with IMRT, patients treated with IMRT were classified as receiving conventionally fractionated radiotherapy. For prostate cancer, the add-on fee was applied when hypofractionation was delivered using IMRT (doses ≥2.5 Gy per fraction from April 2018 to March 2022 and ≥3 Gy per fraction from April 2022 onward). Payment for SBRT was made independently of other external beam radiotherapy techniques. SBRT for prostate cancer was generally used for ultra-hypofractionated regimens (e.g. doses ≥5 Gy per fraction) [5]. Accordingly, radiotherapy for prostate cancer was categorized as conventionally fractionated IMRT, hypofractionated IMRT, SBRT or non-IMRT (other conventional techniques, such as static or moving field irradiation).

This study was approved by the ethics committee of Osaka International Cancer Institute (approval number 24196) and conformed to the provisions of the Declaration of Helsinki. The requirement for patient consent for the CanReCO project was waived because the data were collected for health policy planning and research purposes using an opt-out approach at the participating hospitals.

### Statistical analyses

First, descriptive statistics of annual trends in the proportion of patients who received HF-RT based on the year of diagnosis were analyzed separately for breast and prostate cancers. These trends were further assessed in analyses stratified by facility radiotherapy volume, defined as the median annual number of patients receiving radiotherapy as initial treatment for any cancer, based on each hospital-based cancer registry during the study period. To ensure that each stratum contained similar numbers of eligible patients, facilities were classified as low-volume (<150 patients), medium-volume (150–399 patients) and high-volume (≥400 patients). High-volume facilities included six nationally designated hospitals (one cancer center and five university hospitals). Medium-volume facilities included nine nationally designated and four prefecture-designated hospitals, and low-volume facilities included three nationally designated and 33 prefecture-designated hospitals.

Second, we applied multivariable modified Poisson regression models [19] to explore factors associated with receipt of HF-RT. Relative risks (RRs) and 95% confidence intervals (CIs) were estimated, and statistical significance was defined as a 95% CI that did not include 1.0. Analyses were conducted separately for patients with breast cancer and those with prostate cancer. For each cancer type, models were applied to the overall cohort and to cohorts stratified by diagnosis period (an earlier period [2019–21] and a later period [2022–23]) to assess temporal variations. For patients with breast cancer, those who met the eligibility criteria were included in the analysis. Covariates included facility volume (low, medium or high), age (<50 or ≥50 years), tumor and chemotherapy groups (invasive tumor without chemotherapy, invasive tumor with chemotherapy or noninvasive tumor), laterality (right or left) and receipt of hormone therapy (no or yes). For patients with prostate cancer, the analysis was limited to those treated with IMRT because the add-on fee for hypofractionation was available only for IMRT techniques, and SBRT was generally used for low-risk or a subset of intermediate-risk tumors [5, 20]. Covariates included facility volume (low, medium or high), age (<65 or ≥65 years), clinical TNM classification (cT1–2N0M0 or cT3N0M0), grade group (1–3/unknown or 4–5), and receipt of hormone therapy (no or yes).

All analyses were performed using R software (version 4.2.3; R Foundation for Statistical Computing, Vienna, Austria).

## RESULTS

### Breast cancer

**Fig. 1:**
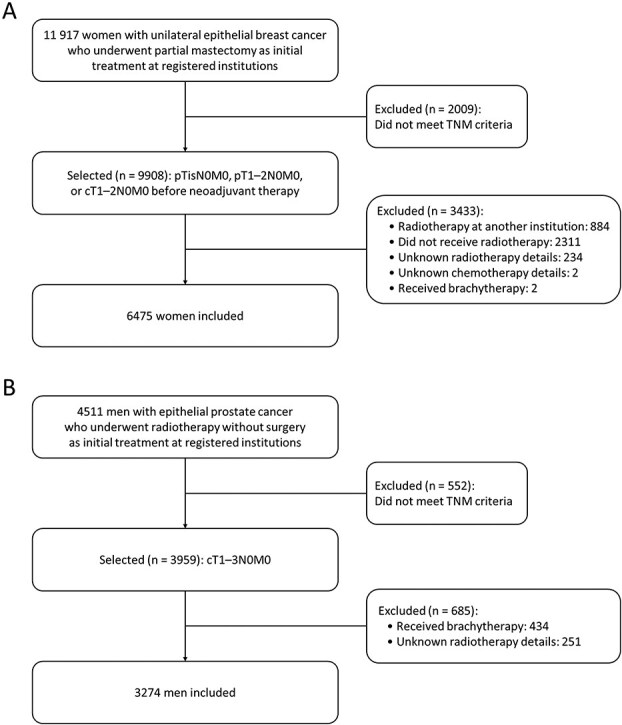
Selection flowchart: (A) patients with breast cancer and (B) patients with prostate cancer. Abbreviation: TNM, tumor-node-metastasis.

A total of 6475 women diagnosed with breast cancer between 2019 and 2023 were included (a selection flowchart is shown in Fig. 1A; patient characteristics are listed in Table 1). The median patient age was 58 years (interquartile range, 49–69 years). Patients were treated at low-, medium- and high-volume facilities in 37%, 30% and 33% of cases, respectively. Regarding tumor characteristics and receipt of chemotherapy, 65% of the patients had invasive tumors and did not receive chemotherapy, 17% had invasive tumors and received chemotherapy, and 18% had non-invasive tumors. Hormone therapy was administered to 70% of the patients.

**Table 1:** Characteristics of patients with breast cancer

Characteristic	Overall, *n* = 6475	Conv., *n* = 2271	Hypo., *n* = 4204
Median age at diagnosis, years	58 (49, 69)	57 (48, 69)	58 (49, 69)
Year of diagnosis			
2019	1328 (20.5%)	784 (34.5%)	544 (12.9%)
2020	1213 (18.7%)	486 (21.4%)	727 (17.3%)
2021	1226 (18.9%)	446 (19.6%)	780 (18.6%)
2022	1385 (21.4%)	306 (13.5%)	1079 (25.7%)
2023	1323 (20.4%)	249 (11.0%)	1074 (25.5%)
Facility volume			
Low	2365 (36.5%)	1363 (60.0%)	1002 (23.8%)
Medium	1971 (30.4%)	537 (23.6%)	1434 (34.1%)
High	2139 (33.0%)	371 (16.3%)	1768 (42.1%)
Tumor and chemotherapy category			
Invasive, no chemotherapy	4229 (65.3%)	1436 (63.2%)	2793 (66.4%)
Invasive, with chemotherapy	1107 (17.1%)	431 (19.0%)	676 (16.1%)
Non-invasive	1139 (17.6%)	404 (17.8%)	735 (17.5%)
Laterality			
Right	3209 (49.6%)	1120 (49.3%)	2089 (49.7%)
Left	3266 (50.4%)	1151 (50.7%)	2115 (50.3%)
Hormone therapy			
No	1972 (30.5%)	777 (34.2%)	1195 (28.4%)
Yes	4503 (69.5%)	1494 (65.8%)	3009 (71.6%)

Data are presented as median (interquartile range) or *n* (%). Facility volume categories were defined by the median annual number of patients who received radiotherapy as initial treatment for any cancer at each institution: low (<150), medium (150–399) and high (≥400). Abbreviations: Conv., conventional fractionation; Hypo., hypofractionation.

**Fig. 2:**
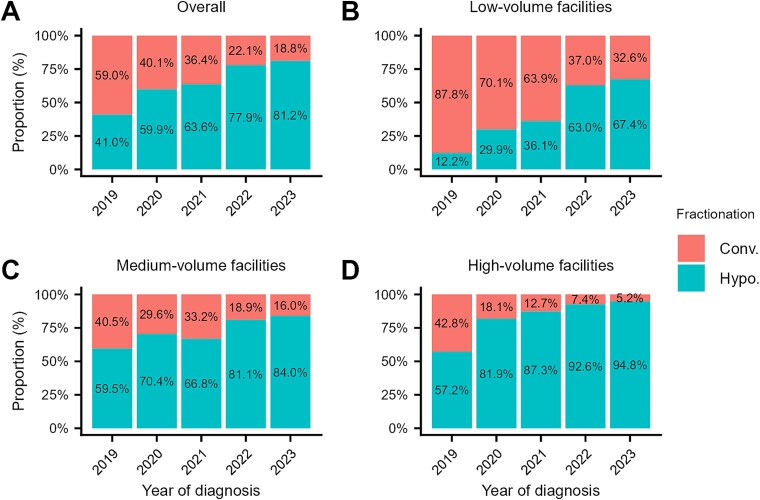
Trends in the proportion of patients with breast cancer who received hypofractionated radiotherapy: (A) all facilities; (B) low-volume facilities; (C) medium-volume facilities; and (D) high-volume facilities. Facility volume categories were defined by the median annual number of patients who received radiotherapy as initial treatment for any cancer at each institution: low (<150), medium (150–399) and high (≥400). Abbreviations: Conv., conventional fractionation; Hypo., hypofractionation.

The proportion of patients receiving HF-RT increased from 41% in 2019 to 81% in 2023 (Fig. 2A). This trend was observed across all facility volume categories (Fig. 2B–D). Among the patients diagnosed in 2023, HF-RT was delivered to 95% of the patients at high-volume facilities, 84% at medium-volume facilities and 67% at low-volume facilities.

**Fig. 3:**
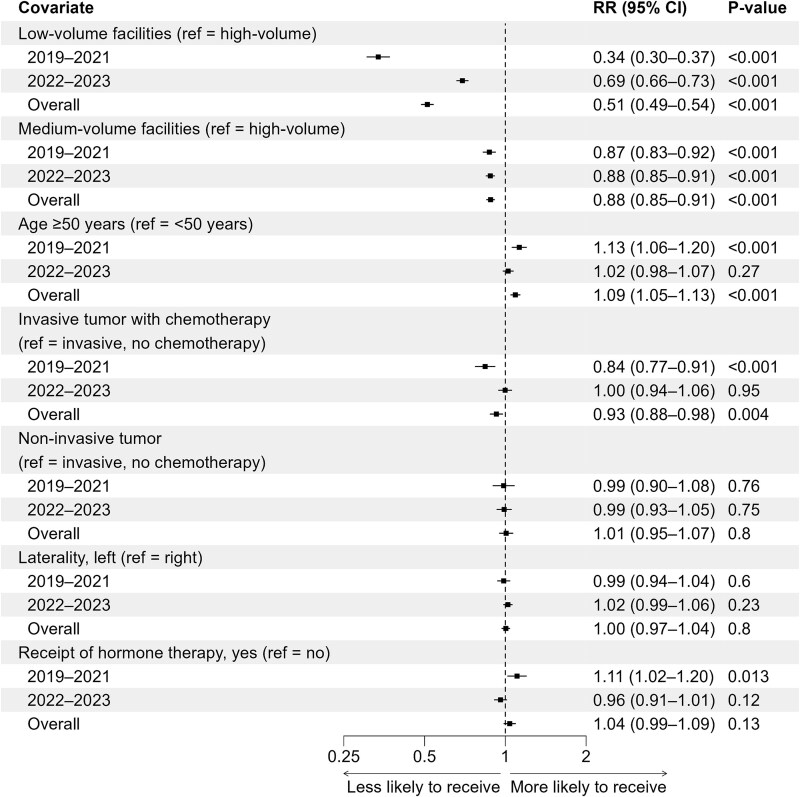
Factors associated with receipt of hypofractionated radiotherapy among patients with breast cancer. Forest plot showing the relative risks and 95% confidence intervals from the multivariable modified Poisson regression analysis in the overall cohort and cohorts stratified by diagnosis period (2019–21 and 2022–23). Abbreviations: CI, confidence interval; ref, reference; RR, relative risk.

Figure 3 presents a forest plot of the RRs and 95% CIs from the multivariable analysis of factors associated with receipt of HF-RT. This analysis included patients with breast cancer in the overall cohort and in subgroups stratified by diagnosis period (2019–21 and 2022–23). Treatment at low- or medium-volume facilities was associated with a lower probability of HF-RT receipt compared with treatment at high-volume facilities (low-volume: RR, 0.51; 95% CI, 0.49–0.54; medium-volume: RR, 0.88; 95% CI, 0.85–0.91). Age ≥50 years was associated with a higher probability of HF-RT receipt compared with age <50 years (RR, 1.09; 95% CI, 1.05–1.13). Among patients with invasive cancer, chemotherapy was associated with a lower probability of HF-RT receipt (RR, 0.93; 95% CI, 0.88–0.98). In 2019–21, hormone therapy was associated with a higher probability of HF-RT receipt (RR, 1.11; 95% CI, 1.02–1.20). In 2022–23, the associations of low-volume facilities, age, chemotherapy and hormone therapy with receipt of HF-RT were attenuated compared with those in 2019–21.

### Prostate cancer

A total of 3274 men with prostate cancer diagnosed between 2019 and 2023 were included (a selection flowchart is shown in Fig. 1B; patient characteristics are listed in Table 2). The median age of the patients at diagnosis was 74 years (interquartile range, 70–78 years). Patients were treated at low-, medium- and high-volume facilities in 29%, 31% and 40% of cases, respectively. Based on the TNM classification, 80% and 20% of patients had cT1–2N0M0 and cT3N0M0 tumors, respectively. The grade group was 1–3 in 60% of the patients and 4–5 in 40%. Hormone therapy was administered to 79% of the patients.

**Table 2:** Characteristics of patients with prostate cancer

Characteristic	Overall, *n* = 3274	Non-IMRT, *n* = 454	Conv. IMRT, *n* = 1533	Hypo. IMRT, *n* = 1103	SBRT, *n* = 184
Median age at diagnosis, years	74 (70, 78)	75 (70, 78)	74 (70, 78)	75 (70, 78)	71 (66, 76)
Year of diagnosis					
2019	699 (21.4%)	117 (25.8%)	406 (26.5%)	139 (12.6%)	37 (20.1%)
2020	609 (18.6%)	87 (19.2%)	308 (20.1%)	182 (16.5%)	32 (17.4%)
2021	625 (19.1%)	71 (15.6%)	274 (17.9%)	241 (21.8%)	39 (21.2%)
2022	684 (20.9%)	89 (19.6%)	291 (19.0%)	266 (24.1%)	38 (20.7%)
2023	657 (20.1%)	90 (19.8%)	254 (16.6%)	275 (24.9%)	38 (20.7%)
Facility volume					
Low	957 (29.2%)	347 (76.4%)	415 (27.1%)	144 (13.1%)	51 (27.7%)
Medium	996 (30.4%)	107 (23.6%)	609 (39.7%)	278 (25.2%)	2 (1.1%)
High	1321 (40.3%)	0 (0.0%)	509 (33.2%)	681 (61.7%)	131 (71.2%)
TNM classification					
cT1–2N0M0	2616 (79.9%)	371 (81.7%)	1141 (74.4%)	923 (83.7%)	181 (98.4%)
cT3N0M0	658 (20.1%)	83 (18.3%)	392 (25.6%)	180 (16.3%)	3 (1.6%)
Grade group					
1–3	1952 (59.6%)	281 (61.9%)	806 (52.6%)	690 (62.6%)	175 (95.1%)
4–5	1308 (40.0%)	169 (37.2%)	721 (47.0%)	409 (37.1%)	9 (4.9%)
Unknown	14 (0.4%)	4 (0.9%)	6 (0.4%)	4 (0.4%)	0 (0.0%)
Hormone therapy					
No	703 (21.5%)	84 (18.5%)	323 (21.1%)	204 (18.5%)	92 (50.0%)
Yes	2571 (78.5%)	370 (81.5%)	1210 (78.9%)	899 (81.5%)	92 (50.0%)

Data are presented as median (interquartile range) or *n* (%). Facility volume categories were defined by the median annual number of patients who received radiotherapy as initial treatment for any cancer at each institution: low (<150), medium (150–399) and high (≥400). Abbreviations: Conv., conventionally fractionated; Hypo., hypofractionated; IMRT, intensity-modulated radiotherapy; SBRT, stereotactic body radiotherapy.

**Fig. 4:**
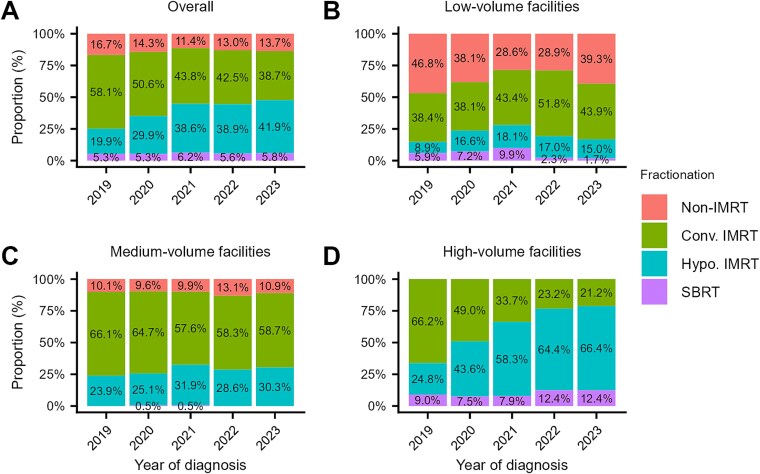
Trends in the proportion of patients with prostate cancer who received hypofractionated IMRT or SBRT: (A) all facilities; (B) low-volume facilities; (C) medium-volume facilities; and (D) high-volume facilities. Facility volume categories were defined by the median annual number of patients who received radiotherapy as initial treatment for any cancer at each institution: low (<150), medium (150–399) and high (≥400). Abbreviations: Conv., conventionally fractionated; Hypo., hypofractionated; IMRT, intensity-modulated radiotherapy; SBRT, stereotactic body radiotherapy.

The proportion of patients receiving hypofractionated IMRT increased from 20% in 2019 to 42% in 2023, although the increase from 2021 to 2023 was modest (an increase of 3 percentage points) (Fig. 4A). The proportion of patients receiving SBRT remained stable at ~5–6% throughout the study period. In total, the proportion of patients receiving hypofractionated IMRT or SBRT increased from 25% in 2019 to 48% in 2023. Regarding facility volume, the use of hypofractionated IMRT or SBRT increased at high-volume facilities, reaching 79% by 2023 (Fig. 4D). The use of hypofractionated IMRT or SBRT at low- and medium-volume facilities increased between 2019 and 2021, and thereafter declined or plateaued, with corresponding proportions of 17% and 30%, respectively, in 2023 (Fig. 4B and C).

**Fig. 5:**
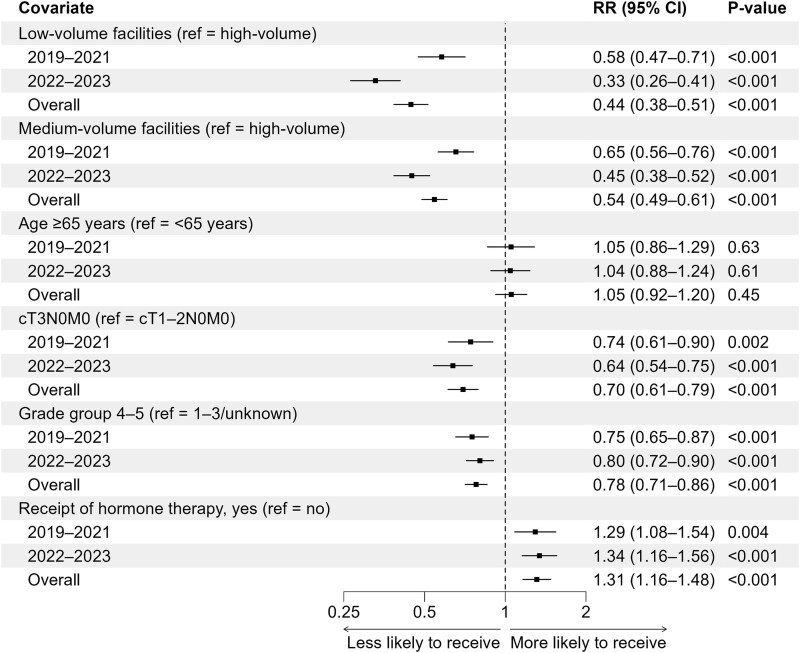
Factors associated with receipt of hypofractionated radiotherapy among patients with prostate cancer who received intensity-modulated radiotherapy. Forest plot showing the relative risks and 95% confidence intervals from the multivariable modified Poisson regression analysis in the overall cohort and cohorts stratified by diagnosis period (2019–21 and 2022–23). Abbreviations: CI, confidence interval; ref, reference; RR, relative risk.

Figure 5 presents a forest plot of the RRs and 95% CIs from the multivariable analysis of factors associated with receipt of HF-RT. This analysis included patients with prostate cancer treated with IMRT in the overall cohort and in subgroups stratified by diagnosis period (2019–21 and 2022–23). Treatment at low- or medium-volume facilities was associated with a lower probability of receiving hypofractionated IMRT compared with treatment at high-volume facilities (low-volume: RR, 0.44; 95% CI, 0.38–0.51; medium-volume: RR, 0.54; 95% CI, 0.49–0.61). Tumors classified as cT3N0M0 or grade group 4–5 were also associated with a lower probability of receiving hypofractionated IMRT (cT3N0M0: RR, 0.70; 95% CI, 0.61–0.79; grade group 4–5: RR, 0.78; 95% CI, 0.71–0.86), whereas hormone therapy was associated with a higher probability (RR, 1.31; 95% CI, 1.16–1.48). In 2022–23, the associations for facility volume were strengthened, and those for tumor stage, grade group and hormone therapy remained stable compared with those in 2019–21.

## DISCUSSION

This study analyzed trends in HF-RT use among patients with breast or prostate cancer in Japan and observed an increase in its use from 2019 to 2023. Among the patients diagnosed in 2023, HF-RT was used in 81% of those with breast cancer and 48% of those with prostate cancer. The observed increase in HF-RT use likely reflects the combined effects of accumulating clinical evidence, supportive guidelines and healthcare system strain during the COVID-19 pandemic [1–6, 10, 11]. However, the extent of this increase differed according to the cancer type and facility radiotherapy volume.

Among the patients with breast cancer, HF-RT use was highest at high-volume facilities, although it increased across all facility volume categories. Among the patients with prostate cancer, hypofractionated IMRT or SBRT use increased from 2019 to 2023 at high-volume facilities, whereas at low- and medium-volume facilities, its use increased until 2021 and plateaued or declined thereafter. The finding that hypofractionation use differed by facility radiotherapy volume is consistent with reports from other regions, where high-volume or academic institutions were more likely to adopt hypofractionation [7–9, 21, 22]. This pattern may be attributable to various factors, including larger teams, peer training and differences in financial incentives [8]. Furthermore, the lack of late-phase increase observed at low- and medium-volume facilities may suggest the presence of certain barriers to adoption.

In a 2018–19 international survey conducted among radiation oncologists [23], the most frequently reported concerns about adopting hypofractionation were insufficient long-term data and potential acute or late toxicity. Although reimbursement systems were infrequently cited as a concern overall, it was more commonly reported in the Asia-Pacific region. In Japan, health insurance payment policies may strongly influence the adoption of hypofractionation. Despite the availability of an add-on fee for hypofractionated regimens, a substantial payment gap remains between hypofractionated and conventionally fractionated IMRT for prostate cancer. For example, as of 2023, the difference in payment between 60 Gy in 20 fractions and 74 Gy in 37 fractions was approximately ¥300 000 (US$2100 based on an exchange rate of ¥140 per dollar). An even larger difference was present between SBRT and conventionally fractionated IMRT. These financial disparities may discourage low- and medium-volume facilities from adopting hypofractionation for prostate cancer treatment. In contrast, the payment gap was smaller for breast cancer treatment, which may support broader hypofractionation adoption, as observed in the present study.

At low-volume facilities, non-IMRT or conventionally fractionated IMRT was predominant in patients with prostate cancer. At medium-volume facilities, conventionally fractionated IMRT was used most frequently. These practice patterns may also reflect institutional and technical constraints. In Japan, facility-level requirements for IMRT include the presence of at least two radiation oncologists. Furthermore, daily image guidance is recommended for safe and effective delivery of HF-RT for prostate cancer [24]. Such requirements increase demands on staffing, equipment and workflow, which may limit the feasibility of HF-RT at smaller facilities.

Among the patients diagnosed with breast cancer between 2019 and 2021, the use of HF-RT was associated with the facility volume, patient age and receipt of chemotherapy or hormone therapy; however, these associations weakened in later years. This change may reflect updates to the breast cancer treatment guidelines. Prior to the 2022 revision, the Japanese guidelines recommended HF-RT for patients aged ≥50 years with pT1–2 N0 tumors who underwent partial mastectomy without systemic chemotherapy, which is in line with the 2011 American Society for Radiation Oncology (ASTRO) guidelines [1, 25]. The 2022 Japanese guidelines expanded this indication to include all patients undergoing partial mastectomy, which is consistent with the 2018 ASTRO guidelines [1, 6]. The observed association between hormone therapy receipt and HF-RT use during 2019–21 suggests that physicians may select hypofractionation based on the molecular subtype of breast cancer. However, multiple studies have shown no significant difference in clinical outcomes between hypofractionated and conventionally fractionated radiotherapy across molecular subtypes [26–28], and current evidence does not support subtype-specific use of hypofractionation.

Among the patients with prostate cancer treated with IMRT, high-risk features such as cT3N0M0 and grade group 4–5 tumors were associated with the non-use of hypofractionation. This pattern aligns with findings from an international survey of radiation oncologists, which reported that hypofractionation was used less often for high-risk than for low- or intermediate-risk prostate cancer [23]. As no clear interaction between treatment efficacy and risk group has been observed, current clinical guidelines support HF-RT even for patients with high-risk tumors [5]. However, patients with high-risk prostate cancer remain underrepresented in major clinical trials [3, 29, 30], which may contribute to physicians’ continued preference for conventional fractionation in this subgroup. Additional clinical evidence may be necessary to encourage the adoption of hypofractionation for these patients. The current study found that the overall adoption of hypofractionation in prostate cancer treatment remained moderate in 2023. The Japanese Clinical Practice Guidelines for Prostate Cancer, revised in 2023, support the use of HF-RT [31]. This revision may encourage its broader use in upcoming years, warranting continued monitoring of trends.

The receipt of hormone therapy was associated with a higher use of hypofractionation among patients with prostate cancer. One possible explanation for this is physicians’ concern about radiotherapy-related toxicity in patients not receiving hormone therapy, who often have larger prostate volumes. Hormone therapy reduces the prostate size and may lower the incidence of rectal toxicities when administered before radiotherapy [32]. To date, there is no evidence that hypofractionation compared to conventional fractionation increases toxicity in patients with large prostate volumes [5]. This suggests that the absence of hormone therapy is an insufficient reason to avoid HF-RT.

A key strength of this study was the inclusion of a multicenter cohort, which covered the majority of patients with breast or prostate cancer in Osaka Prefecture, to characterize practice patterns of HF-RT use. The findings indicated that further investigation regarding the barriers to HF-RT implementation may be required to promote its broader adoption. However, this study had certain limitations. First, the analysis was limited to institutions in a single prefecture, Osaka. Although payments to healthcare providers are standardized nationwide, regional differences in the use of HF-RT may exist. Further research is warranted to evaluate national trends and assess regional variations in its use. Second, information on dose fractionation was unavailable; thus, specific dose fractionation schedules could not be identified. Consequently, this study could not distinguish between moderate and ultra-hypofractionation in breast cancer radiotherapy. In addition, an add-on fee for HF-RT in breast cancer treatment cannot be claimed when patients receive IMRT. Accordingly, all patients who received IMRT were classified as having undergone conventionally fractionated radiotherapy. However, in this study, only 33 patients with breast cancer received IMRT (data not shown in the Results section), and the potential impact of this limitation is likely to be small. In prostate cancer treatment, an add-on fee for HF-RT can be claimed when the treatment is billed as IMRT. Furthermore, in April 2022, the criteria for this add-on fee were revised to allow the fee when the dose per fraction increased from ≥2.5 to ≥3 Gy. Accordingly, patients who received HF-RT without an IMRT claim or those treated from April 2022 onward with a hypofractionated regimen at doses <3 Gy per fraction could not be identified. This may have led to an underestimation of the proportion of patients receiving HF-RT. Finally, the patients were stratified according to diagnosis year. There may have been a delay between diagnosis and the receipt of radiotherapy, particularly among patients with breast cancer receiving chemotherapy and those with prostate cancer receiving hormone therapy. This potential delay could have led patients to receive radiotherapy in a later period, thereby biasing the estimated association toward greater use of hypofractionation.

In conclusion, the use of HF-RT has increased among patients with breast and prostate cancer in Osaka, Japan. Nevertheless, its adoption in prostate cancer treatment remains limited, particularly at low- and medium-volume facilities. These findings suggest that specific barriers may hinder HF-RT adoption and highlight the need to identify and address these factors.

## Data Availability

Data were provided by participating hospitals under a confidentiality agreement and are not publicly available.
